# Review of Two Popular Eating Plans within the Multiple Sclerosis Community: Low Saturated Fat and Modified Paleolithic

**DOI:** 10.3390/nu11020352

**Published:** 2019-02-07

**Authors:** Terry L. Wahls, Catherine A. Chenard, Linda G. Snetselaar

**Affiliations:** 1 Department of Internal Medicine, Carver College of Medicine, University of Iowa, Iowa City, IA 52242, USA; catherine-chenard@uiowa.edu; 2 Department of Epidemiology, College of Public Health, University of Iowa, Iowa City, IA 52242, USA; linda-snetselaar@uiowa.edu

**Keywords:** low saturated fat diet, Paleolithic diet, multiple sclerosis, modified Paleolithic diet, Swank diet, Wahls diet, Wahls Elimination diet

## Abstract

The precise etiology of multiple sclerosis (MS) is unknown but epidemiologic evidence suggests this immune-mediated, neurodegenerative condition is the result of a complex interaction between genes and lifetime environmental exposures. Diet choices are modifiable environmental factors that may influence MS disease activity. Two diets promoted for MS, low saturated fat Swank and modified Paleolithic Wahls Elimination (WahlsElim), are currently being investigated for their effect on MS-related fatigue and quality of life (NCT02914964). Dr. Swank theorized restriction of saturated fat would reduce vascular dysfunction in the central nervous system (CNS). Dr. Wahls initially theorized that detailed guidance to increase intake of specific foodstuffs would facilitate increased intake of nutrients key to neuronal health (Wahls™ diet). Dr. Wahls further theorized restriction of lectins would reduce intestinal permeability and CNS inflammation (WahlsElim version). The purpose of this paper is to review the published research of the low saturated fat (Swank) and the modified Paleolithic (Wahls™) diets and the rationale for the structure of the Swank diet and low lectin version of the Wahls™ diet (WahlsElim) being investigated in the clinical trial.

## 1. Introduction

Multiple Sclerosis (MS) is a chronic, inflammatory, immune-mediated condition that damages nerve fibers and the myelin sheath and affects the brain, spinal cord and optic nerve [1]. The cause of MS is unknown, however, it is believed to be a result of complex interaction between genetic and environmental exposures, including diet [2]. Treatment consists of disease-modifying therapies [1,3], symptom and relapse management and support from physical and occupational therapists [4] and dietitians [5]. Clinical disease course is unpredictable and may result in significant disability including unemployment due to disease progression [6,7,8]. 

Because modifiable lifestyle factors such as diet quality may impact the disease course [7,8,9,10,11], dietary guidelines for persons with MS (pwMS) have the potential to reduce MS-related symptoms. Potential mechanisms by which diet quality may influence disease course in MS patients include epigenetic changes in gene expression [12] and shifts in the composition of the gut microbiome [13] both of which may result in down regulation of inflammation. Diet quality may also influence the sufficiency of nutrients required for neuronal structure [14,15].

PwMS frequently use [16] or express interest in using [17] a variety of special diets to try to treat their disease [18]. Examples include the low saturated fat Swank [19,20,21,22,23,24,25,26], plant-based low-fat McDougall [27], Mediterranean [11,28], ketogenic [29], energy restriction/fasting [30,31,32] and modified Paleolithic (Paleo) Wahls™ [33,34,35,36,37] diets. However, clear evidence to support the benefit of any specific dietary regimen is lacking and well-designed, randomized controlled trials are needed [38,39]. Because there is not currently enough evidence to recommend a specific diet for MS, the National MS Society (NMSS) [40,41] recommends pwMS follow healthy eating guidelines for the general population such as the U.S. Dietary Guidelines for Americans (DGA) [42] and those for cancer and heart disease prevention [43,44]. To address the demand for research on the safety and efficacy of diets promoted for MS, a randomized clinical trial is currently underway [45,46] to compare the effect of two diets, Swank and Wahls Elimination (WahlsElim), on MS-related fatigue. 

These diets were selected for comparison because they were specifically designed to treat MS and are popular within the MS community [40]. The study protocol for the clinical trial comparing Swank and WahlsElim diets has been reported [45,46]. Data from previous studies allowed us to perform power calculations to detect clinically and statistically significant changes in fatigue measured by the fatigue severity scale [35] and quality of life (QOL) measured by the MS Quality of Life 54 [46] questionnaire over a 12 week period; this allowed for a shorter intervention period and less costly study design. The study has three 12 week periods for a total of 36 weeks. The first 12 weeks is an observation period where participants continue to eat their usual diet. They are then randomized and trained on the study diet by a Registered Dietitian (RD) and begin the second 12 week period; participants receive ongoing support from a RD for adopting and sustaining the assigned study diet for 12 weeks. The third 12 week period is to assess how well participants sustain the study diet when they are not receiving ongoing support from the study team. Assessments including fatigue, QOL and diet quality are completed twice during the observation period (baseline and 12 weeks) and twice during the intervention period (24 and 36 weeks). The initial 12 week observation period allows participants to serve as their own control. We will assess whether adopting either diet is associated with reduced fatigue and improved QOL after 12 weeks on the diet and also whether one diet has superior efficacy at reducing perceived fatigue or improving QOL. Fatigue is the main outcome measure because it is a common complaint of pwMS which results in reduced QOL and for which there is no effective pharmacological treatment [47]. 

The purpose of this paper is to provide an overview of the published research on the use of the Swank and Wahls™ diets and rationale for the structures of the Swank and WahlsElim diets being investigated in the Dietary Approaches to Treat Multiple Sclerosis Related Fatigue Study (NCT02914964) [45]. The two diets are described in Table 1.

## 2. Swank Low-Saturated Fat Diet Development and Research

The Swank diet is a low-fat, low-saturated fat eating pattern developed in 1948 by Dr. Roy Swank based on epidemiological evidence that increased consumption of fat, especially from meat and dairy, was associated with higher incidence of MS [19,20]. Swank initially recommended his patients consume 20–30 g fat per day and revised this to ≤20 g saturated fat [20] and then ≤15 g in 1952 [21]. He also recommended 5 g cod liver oil, 10–15 g vegetable or fish oil, one egg, a multivitamin, whole wheat bread [20] and fish ≥3 times per week [23]. In later years his patients’ oil consumption increased to 40–50 g per day [23]. The diet also included an additional 10 g of fat from other sources such as bread [23]. The precepts of the Swank diet are described in *The Multiple Sclerosis Diet Book* [48] published in 1987. The Swank MS Foundation and website [49] are now the primary sources of information about the diet since Dr. Swank’s death in 2008 [50]. 

In addition to the diet, Swank recommended his patients obtain adequate rest by lying down during the middle of the day for 30 min to two hours, reduce stress and keep a positive outlook ([48] pp. 3, 55). Recent research has shown stress reduction and mindfulness beneficial in reducing fatigue and improving QOL in pwMS [51,52,53,54]. 

Swank published a series of reports from 1953 to 2003 following a cohort of 150 patients at the Montreal Neurologic Hospital who were diagnosed with relapsing (exacerbating) remitting MS and treated with a low saturated fat diet. In his initial report [20], 150 patients with MS had been trained on a low-fat diet and maintained continuous contact with the hospital. Of these 150 patients, 47 continued on the low saturated fat diet for more than two years. Patients were trained on the low saturated fat diet by RDs and were given notebooks to record their daily food intake. Patients met with RDs initially every two weeks, then monthly and then annually to provide additional instructions and support as needed. Data from the 47 patients was reported as a single arm cohort study in the initial report. The three years prior to the initial assessment and training in the low saturated fat diet was a control period, and the three years after the initial assessment was the intervention period. Age at diagnosis and age at onset of low-fat diet were recorded. Patients were interviewed and hospital records reviewed to assess the frequency and severity of MS attacks. Severity of MS attacks was rated numerically 1 to 4 (most severe). Performance status was recorded yes or no for the following categories: chair, walking, working part time and working full time. Change in performance while on low fat diet was recorded as unchanged, improved or deteriorated. Lipid intake to the nearest five grams of fat and oil intake and regular or irregular pattern of following the low saturated fat diet at the most recent visit were noted. Forty-one of the 47 patients were walking and working full time when they were placed on the diet. Exacerbations were less frequent and less severe while on the low saturated fat diet [20]. The patients with more advanced disability and in the progressive phase of the illness continued to decline.

In his next paper, Swank reported four to five and one half years of follow up of the original cohort of 47 patients [21]. Three patients had been lost to follow up. He again reported lipid intake, performance status, number and severity of exacerbations. He again noted patients who were most compliant with restricting saturated fat intake had fewer and less severe exacerbations. Those with more disability or in the progressive phase of MS were likely to experience continued decline even if compliant with the diet. [21] 

The next paper was a seven year follow up [22]. The original cohort was combined with an additional 107 for a total of 153 MS patients followed by the Montreal Neurologic Hospital, the Veteran Administration Hospitals in Canada or New York State or Dr. Roy Swank. All patients had been instructed on and were continuing to follow a low saturated fat diet. Again, excerebrations were less frequent and less severe in the patients most adherent to the low saturated fat diet [22]. In the nine year report, 32 of the previous 153 patients were following the dietary prescription poorly and were excluded, leaving 121 patients in the analysis [55]. Again, exacerbations were less frequent and less severe in the patients who had severely restricted their saturated fat intake. 

At the 20 year report, several patients had been lost to follow up, leaving a cohort of 146 patients for analysis [23]. Patients recorded all foods consumed in the week prior to their clinic visits. After July 1954 patients sent food records every three months through the 15th year of the study and then annually. Diet was assessed by two observers. Patient performance was assessed annually. Eighty percent of patients were categorized by Swank as ‘good dieters’ (consuming ≤20 g of fat) with the other 20% classified as ‘poor dieters’ (consuming >20 g of fat). Good dieters had fewer and less severe exacerbations and individuals with greater disability were more likely to experience progressive worsening [23].

At 34 years, two more patients had been lost to follow up, leaving a cohort of 144 patients for analysis [24]. Cause of death was identified and classified as MS-related or not MS-related. There were 81 deaths and 63 living patients who came in for examination. Again those who consumed ≤20 g of fat were less likely to have died (MS-related or not MS-related) and experienced fewer and less severe exacerbations and were more likely to still be walking [24].

In 2000 Swank contacted patients in the good dieting group who were age 72 to 84 years via mail and a study team member was able to complete a home visit with 15 of these patients. Thirteen of the 15 were still ambulating, living at home and caring for themselves [26]. This was Swank’s last report of the cohort he followed for 50 years.

Swank believed MS had a vascular component [20]. His diet is consistent with the cholesterol hypothesis of atherosclerosis that suggests saturated fat has deleterious effect on cerebrovascular health [56,57]. Recent studies by other investigators found positive associations between worsening disability and total cholesterol and triglycerides levels and negative associations between HDL cholesterol and disability status of pwMS [58,59]. Vascular comorbidities have also been associated with increased disability [60]. Higher energy intake from fat, especially saturated fat, was associated with increased risk of relapse in a study of 219 children with MS [61,62]. 

Swank’s studies have been criticized for not being randomized controlled trials; his comparison of ‘good’ and ‘poor’ responders may also be biased towards a positive result because patients who are feeling well are more likely to adhere to the ≤20 g saturated fat restriction while those who are declining may abandon it [63]. His reports were also criticized for not using blinded assessors [64,65]. Additional weaknesses include the risk of missing data that is not missing at random but missing as part of the disease process, selection bias, exclusion of patients who were following the diet ‘poorly,’ absence of brain MRI data and the lack of standardized validated dietary assessment measures. The strength of the Swank cohort study is long duration of the follow up period and size of the cohort. 

Despite being developed 70 years ago, the Swank diet is still in use. In 2003, prior to the development of the Wahls™ diet, a survey of complementary and alternative medicine (CAM) use in 3140 individuals in the United States with MS found 16% of respondents following a Swank diet compared with 10.4% who were making other dietary changes. Mean (SD) perceived effectiveness of Swank compared to other diets was 3.16 (1.67) versus 2.7 (1.66), respectively, based on a six point scale from 0 (not effective) to 5 (very effective); however, results were limited by the low (27.1%) response rate [66]. A small 2015 CAM survey of 35 pwMS reported 27/35 (77.1%) using CAM and 7.4% (n = 2) individuals following the Swank diet [67]. A 2018 report of 6989 pwMS found 6.7% of individuals were following the Swank diet now or in the past [68]. 

## 3. Wahls Elimination Diet Development and Research

The modified Paleolithic (Paleo) Wahls™ diet was originally developed in 2008 by Dr. Terry Wahls based on the tenets of a Paleo diet [69,70,71] and her review of the scientific literature [72,73]. Paleo diets which exclude grains, dairy and legumes and emphasize fruits and vegetables (F/V), meat and fish are nutritious [70,71] and have been associated with improved biomarkers associated with reduced risk for heart disease [74] such as reduced blood pressure, weight, waist: hip ratio, improved lipids, increased insulin sensitivity, decreased liver fat and increased satiety [75,76,77,78,79,80,81,82]. An observational survey of psoriasis patients following a Paleo diet reported an association with reduced skin symptoms and improved QOL [83]. 

The modified Paleo diet (Wahls™) was first reported in a case report which documented significant regression of disability from wheelchair dependence to mild gait disability using a modified Paleolithic diet, exercise, electrical stimulation and supplements as part of a multimodal lifestyle intervention (T.W.) [73]. 

A subsequent multimodal single-arm, open-label, safety and feasibility study of the modified Paleolithic (Wahls™) diet plus dietary supplements, electrical muscle stimulation, exercise, meditation and self-massage showed a reduction in fatigue and increase in QOL over 12 months for a group of 20 individuals with progressive MS [33,34]. Individuals who showed the largest reduction in fatigue had less disability at baseline [34]. Participants with mild-moderate gait impairment showed a clinically significant improvement in 25-foot walking speed while those with more significant impairment were less likely to experience meaningful improvements in gait [37]. The decrease in fatigue at three months was associated with a faster walking speed at 12 months [37]. Improvements were also seen in mood and cognitive function which were associated with improved fatigue; those who adhered more closely to the multimodal intervention saw greater improvements [36]. Improvement in lipid profile with a reduction in triglycerides and improvement in high density lipoprotein cholesterol were associated with a reduction in fatigue severity [84]. Dietary intake assessed by the Harvard Food Frequency questionnaire (FFQ) at baseline and end of study showed a reduced energy intake at end of study [84]; percent energy from total fat, monounsaturated and polyunsaturated fats, protein and natural sugar increased and percent Calories from carbohydrate, added sugar, saturated and *trans*-fat decreased. Dietary fiber intake increased as did omega-3 and omega-6 fatty acid intake; glycemic index and load were reduced. 

The limitations of this pilot study were the lack of randomization, the multimodal nature of the intervention, variability in supplement intake, lack of blinded assessors and brain MRI data and small study size. Micronutrient intake from the FFQ and nutrient data from 24 h dietary recalls collected at end of study have not yet been reported.

To investigate the potential impact of the diet alone, a small (n = 17) randomized, waitlist controlled trial of the modified Paleolithic (Wahls™) diet was conducted [35]. Individuals with relapsing-remitting MS were studied because data from the pilot study suggested that pwMS who have less disability may be more responsive to the diet. Fatigue and QOL improved after three months on the Wahls™ diet compared to participants randomized to their usual diet [35]. Study strengths include randomization and use of a control group. Study limitations included small sample size, short intervention duration, 35% dropout rate, lack of assessor blinding and the higher economic status of participants (subjects stated they were able to accommodate a potential 30% increase in grocery expenses).

The original modified Paleolithic (Wahls™) diet [33,85] used in these studies differs from a typical Paleo diet in regard to the recommendation for nine+ cups (~1950+ grams) F/V divided among leafy, sulfur-rich and deeply colored; encouragement of seaweed, algae and nutritional yeast consumption; allowance for limited servings of gluten-free grains (e.g., rice) and legumes (e.g., soy milk); elimination of eggs which are normally allowed on a Paleo diet; and lower meat/fish intake. In 2015 Dr. Wahls developed the Wahls Elimination (WahlsElim) diet, eliminating gluten-free grains, legumes and nightshades (e.g., tomatoes, white potatoes, eggplant, peppers and seed spices) to reflect the theoretical benefits of a low lectin diet [86,87,88]. Lectins are associated with increased intestinal permeability and increased innate immune cell activation, which may be associated with increased symptoms in rheumatoid arthritis patients [86]. A single arm pilot study of a low lectin Paleolithic diet eliminating foods believed to cause inflammation was associated with favorable clinical response documented by biomarkers and endoscopy score in individuals with inflammatory bowel disease [89]. 

## 4. Rationale for the Structure of the Swank and Wahls Elimination Diets

### 4.1. Calories (Kilojoules)

Obesity during childhood increases the risk of developing MS [90,91]. The European Society for Parenteral and Enteral Nutrition (ESPEN) guideline for clinical nutrition in neurology recommends preventing obesity in adolescence and young adulthood for the prevention of MS [92]. Elevated BMI has been associated with increased disability and risk of relapse [93]. Obesity also increases the risk of brain volume loss once MS has developed [94]. Individuals with MS may also be at risk for malnutrition, especially individuals with dysphagia who have difficulty consuming adequate energy and nutrition; the ESPEN recommends routine screening for dysphagia and early detection and treatment of malnutrition in pwMS [92]. The 2015–2020 Dietary Guidelines for Americans (DGA) which provides advice for consuming a healthy diet to reduce risk for diet-related chronic diseases also recommends individuals consume energy appropriate for attaining a healthy weight [42]. 

Swank advised his patients to consume carbohydrate in sufficient quantity to meet energy needs, however, he observed that his patients lost weight because of reduced energy intake [23] and underweight patients had better outcomes [20]. Their weight was typically 5–10% below normal and Swank encouraged them to maintain the lower weight [55]. Dr. Wahls also recommends individuals consume sufficient energy to achieve and maintain a normal body mass index (BMI). However, despite advising participants to consume adequate energy she also observed weight loss among pwMS following her diet for 12 months which resulted in an average 7.7% (+1.8 to −23.9%) kg/m^2^ reduction in BMI [33]. Neither diet recommends fasting. 

### 4.2. Fruits and Vegetables

Fruits and vegetables (F/V) have high nutrient density and are associated with decreased total and cardiovascular mortality [95]. Low micronutrient intake may be associated with higher rates of neurodegeneration [96]. The DGA recommends consuming vegetables daily and specific quantities of dark-green, red/orange, beans/peas, starchy (e.g., white potatoes, peas, corn) and other vegetables (e.g., green beans, onions, iceberg lettuce, celery, cabbage) throughout the week [42]. The DGA Healthy US-Style Pattern (HEP) also recommends daily consumption of one and a half to two and a half or more cup-equivalents of fruit, especially whole fruit, per day depending on energy level [42]. There is no specific F/V recommendations for pwMS beyond the DGA, however, a pediatric MS study found risk for relapse reduced by 50% with a one cup increase in vegetables [62].

F/V are allowed in unlimited quantities on the Swank diet with the minimum recommended vegetable amount similar to the 1600 kcal (6694 kJ) DGA HEP but less than suggested for higher energy levels [42]. However, specific recommendations for amounts of dark-green, red/orange and other vegetables subgroups are not provided. The DGA available in 1987, the year Dr. Swank published *The Multiple Sclerosis Diet Book. A Low-Fat Diet for the Treatment of M.S.* [48], included general advice to consume a variety of foods but no recommendations for quantities of specific F/V subgroups [97]. High fat vegetables such as avocado and olives are counted towards the oil intake on the Swank diet (see Section 4.6.3. Monounsaturated and Polyunsaturated Fatty Acids).

The WahlsElim diet recommends nine or more servings of F/V per day, more than the DGA HEP, and in a very specific pattern of two to three+ servings each of green leafy vegetables, sulfur-rich vegetables and deeply pigmented F/V with a goal of 200 or more different plant species per year. The additional spices, herbs and plant species increases phytochemical diversity and may reduce inappropriate and excessive inflammation [98,99]. Eating more non-starchy vegetables, resistant starch and soluble fiber plus fermented foods from allowable foodstuffs (see Section 4.12.3. Fermented Food) are recommended with the goal of passing one to two soft bowel movements daily to reduce constipation, a common symptom [100] in pwMS.

Dark-green leafy vegetables, an important recommended F/V component of the WahlsElim diet, are excellent sources of vitamin K and carotenoids. The recommended numbers of dark green leafy servings are greater than the DGA HEP amounts. Vitamin K is involved in sphingolipid metabolism, cell membranes, enhances remyelination and oligodendrocyte precursor cells [101,102,103,104]. Vitamin K is metabolized by gut bacteria into vitamin K2mk7 which facilitates absorption of ectopic calcium from blood vessels back into the blood stream and mineralization of teeth and bones [105,106]; enhanced absorption may be especially important on the WahlsElim diet because dairy products, a good source of calcium, are eliminated and the diet may be low in this nutrient. Carotenoids are precursors to retinol (vitamin A) which improves the balance between Th17 cells and T Regulatory cells. Retinol is considered an important target in the control of MS disease activity [107,108]. β-carotene is converted to vitamin A in the intestine by the enzyme β-carotene-15,15’-monoxygenase (BCMO1) to support vision, reproduction, immune function and cell differentiation. However, considerable variability in BCMO1 exists and can affect individual vitamin A status with some single nucleotide polymorphism (SNP) variations having a 69% decrease in enzymatic efficiency [109,110]. Therefore, the diet also includes other vitamin A sources in retinol (preformed vitamin A) from organ meat (see Section 4.3.2. Organ Meat) and cod liver oil (see Section 4.13.1. Cod Liver Oil). 

Cabbage and onion family vegetables and culinary mushrooms are another recommended vegetable category on the WahlsElim diet. These foods are rich sources of organic sulfur. Sulfur may protect from neuro inflammation and/or neurodegeneration and provide favorable modulation of immune cell function [111,112,113]. Diets high in sulfur may also reduce the risk of heavy metal toxicity [114]. Culinary mushrooms have a long history of medicinal use as favorable modulators of immune response [115,116,117,118]. Lion’s mane mushroom has been associated with increased production of nerve growth factors [119].

Deeply pigmented F/V are the third recommended F/V category on the WahlsElim diet. These foods, especially berries, are associated with higher polyphenol and antioxidant content [120,121,122,123,124,125] which is associated with decreased risk of cognitive decline [126,127] and neurodegeneration [128,129]; higher polyphenol phytochemical content may be anti-inflammatory and immune modulating [99]. A variety of colors are encouraged on the WahlsElim diet: red, yellow/orange, blue/black/purple and green.

On the WahlsElim diet, white F/V (e.g., potatoes, bananas, apples, pears) consumption is discouraged until leafy, sulfur and colored F/V guidelines are met. Although white F/V provide dietary fiber and other beneficial components [130,131,132,133], these F/V are often preferentially consumed instead of deeply colored F/V. Delaying consumption of white F/V is a dietary strategy to help promote consumption of leafy, sulfur and deeply colored F/V. Although apples have colored skin, the majority of the apple is white and the fruit is therefore classified as “white”; by contrast, “colored” F/V are colored throughout. Two other white vegetables, onions and cauliflower, are included in the sulfur-rich group. White potatoes are also excluded because they are nightshades.

Nightshades are avoided on the WahlsElim diet. White potatoes, tomatoes, eggplant and peppers are members of the nightshade (Solanaceae) family. Nightshades contain lectins and alkaloids, which may cross react with autoantibodies and increase disease activity in systemic autoimmune disease [86,87,88]. These lectins and alkaloids may negatively disturb nerve, brain, muscle and digestive functions in the body. Eliminating nightshades may decrease inflammation as well as lower pain in joints and muscles and reduce brain symptoms. Lectins are found in wheat, legumes, nuts, seeds and white potatoes and are associated with an abnormal immune response in genetically vulnerable individuals [86,87,88]. Susceptible individuals who consume large amounts of lectins may experience damage to their gut lining causing increased intestinal permeability which may allow bacterial fragments such as lipopolysaccharide (LPS) and incompletely digested food proteins to enter the blood stream. These protein fragments may trigger an immune response potentially culminating in autoimmune disease. Increased intestinal permeability is seen in MS [134,135]. It has been proposed that greatly reducing lectin load may reduce symptoms in some susceptible individuals [86,87,88]. Soaking grains, nuts, seeds and legumes will reduce the lectin content; using high pressure cooking for nightshades, grains and legumes will denature the lectin, making the foods less inflammatory but will not denature gluten and may not denature casein [136]. Fermentation is another option for reducing lectins [137]. Nightshade foods may be re-introduced to the WahlsElim diet one ingredient at a time after three months; these foods may be consumed if no increase in neurological or medical symptoms is observed in the week following reintroduction.

### 4.3. Protein Foods

The DGA recommend consuming five ounce-equivalents (142 g) of protein foods per day for the 1600–1800 kcal (6694–7531 kJ) Healthy US-Style Pattern (HEP) diets and a variety of protein types over the course of a week including meat, poultry, eggs, seafood, nuts, seeds, beans and peas and soy [42]. Eight ounces (227 g) of seafood is recommended per week for 1600–2000 kcal (6684–8368 kJ) diets and four ounces (113 g) nuts, seeds and soy per week for 1600–1800 kcal (6684–7531 kj) diets [138]. Healthy vegetarian diet patterns are also available in the DGA which exclude meat and poultry [42,139]. 

There are no specific protein recommendations for pwMS beyond those for the healthy population. However, some limited evidence shows potential benefit for consumption of fatty fish. A pilot study of patients with mild ulcerative colitis eating 600 g of Atlantic salmon per week showed reduced inflammatory bowel disease activity after 8 weeks [140]. An Australian case-control study found a higher intake of omega-3 fatty acids from fish associated with reduced risk of first demyelinating event although fat from supplements was not assessed [141]. Alternatively, one physician (McDougall) advocates a vegan low fat diet to treat MS that emphasizes starchy foods (e.g., grains, tubers, legumes) plus vegetables and fruit [142]. A 12 month randomized trial investigating this vegetarian (no meat, fish, eggs, dairy or vegetable oils) low (14%) fat diet compared to a usual diet (~40% fat) found improvements in fatigue, weight and blood lipids but no change in disease activity [27]. 

The Swank and WahlsElim diets are not vegetarian diets, however, the amount and types of protein foods vary between the diets as described below. Swank did not clearly prescribe a daily target protein amount other than it should be of adequate “quantity and quality” ([48] p. 126); one of his early publications recommended 50 g or more protein per day [20] and his book referenced 1.0 g protein/kg body weight ([48] p. 101). Protein containing foods are not restricted on the Swank diet except for those imposed by the requirement to limit saturated fat to ≤15 g per day. The WahlsElim diet does not prescribe a specific total protein goal but the minimum 6 ounce (170 g) recommendation for meat/fish intake alone (Table 1) meets or exceeds the DGA recommendation for diets with ≤2200 kcal (9205 kJ) and provides approximately 42 g protein, with additional protein contributed by nuts and vegetables. 

#### 4.3.1. Beef/Pork/Poultry/Fish

The Swank diet excludes beef, pork, organ meat, dark meat poultry and poultry skin during the first year because of the higher saturated fat content. Swank recognized the beneficial components in fatty fish and included them in the diet in limited amounts that kept the diet within the fat guidelines. Fat from fatty fish are counted toward oil intake (see Section 4.6.3. Monounsaturated and Polyunsaturated Fatty Acids). 

Meat and fish are recommended for the WahlsElim diet because they contain all essential amino acids and most other sources of protein are eliminated on this diet (e.g., grains, legumes, dairy, eggs). However, the recommended meat and fish intake is lower than most Paleo diets to reduce the activity of mammalian target of rapamycin (mTOR), insulin and insulin-like growth factor [143]. High levels of insulin are associated with higher risk of insulin resistance, metabolic syndrome and obesity [144]. High levels of mTOR are associated with imbalance of T cell homeostasis and increase of autoimmunity [145]. Inhibition of insulin-like growth factor is a potential target of therapy for another autoimmune condition, rheumatoid arthritis [146]. 

Grass fed and finished meats, wild game and wild caught fish and shellfish are also encouraged on the WahlsElim diet within the financial means of the individual to increase intake of eicosapentaenoic acid (EPA) and docosahexaenoic acid (DHA) (see Section 4.6.3. Monounsaturated and Polyunsaturated Fatty Acids). Although fatty fish are better sources of omega-3 fatty acids than meat, grass fed animals have higher amounts of omega 3 fatty acids than those that are grain fed [147] which provide a more favorable omega 6:omega 3 ratio (see Section 4.6. Fats). The WahlsElim diet recommends 16 ounces (454 g) fatty fish per week, more than included in the HEP.

#### 4.3.2. Organ Meat

Organ meat is not specifically mentioned in the DGA but it is included as part of their meat subgroup [42]. Organ meat is not allowed on the Swank diet during the first year due to the high saturated fat content; limited amounts of 3 ounces (85 g) per week are allowed after one year on the Swank diet [48]. The WahlsElim diet encourages consumption of organ meats, especially liver, to increase nutrient intake and prevent deficiency. They are good to excellent source of B vitamins, vitamins A and D, phosphorus, zinc, selenium, copper and manganese. Liver is an excellent source of retinol (preformed Vitamin A) which is an important nutrient in bone metabolism, immune cell function, retinal health and cell differentiation. It is also an excellent source of choline and coenzyme Q10 [148]. 

#### 4.3.3. Eggs

Swank allows an unrestricted amount of egg white but limits whole eggs to three per week because of the high saturated fat content of the yolk [48]. Eggs are normally allowed on a Paleo diet [149] but they are avoided on the WahlsElim diet because eggs are a common food allergen, especially in children [150]. More than 70% of individuals with inflammatory bowel disease react to egg albumin [151], 60% of seasonal allergy patients have IgG antibodies to eggs [152] and 13% of adults with seasonal allergy have IgG antibodies to eggs [153]. Eggs along with wheat and milk are common food triggers to Eosinophilic Esophagitis [154].

#### 4.3.4. Nuts and Seeds

Swank recommends nuts for snacks [48,49]. Because of the high fat content, nuts and seeds must be counted towards oil intake on the Swank diet (see Section 4.6. Fats). 

The quantity of nuts and seeds is limited to four ounces (113 g) per day on the WahlsElim diet to avoid excess energy intake and provide an omega 6:3 ratio approximately 4:1 [155]. This maximum daily quantity of nuts is higher than the weekly DGA recommendation for nuts, seeds and soy on the HEP [138]. Traditional preparation of nuts and seeds often utilize soaking and sprouting which increases enzyme activity and reduces lectin activity [136]. Soaking begins the germination phase which reduces the lectin content thereby reducing the probability of an abnormal and excessive immune response [156]. For this reason, the WahlsElim diet recommends soaking nuts and seeds for 6 to 24 hours and then rinsing to reduce lectin content (see Nightshades in Section 4.2. Fruits and Vegetables); individuals should consume soaked nuts/seeds immediately or dry in a dehydrator or oven for later use. 

#### 4.3.5. Legumes

Legumes (e.g., dried beans, peas, green beans, soybeans, soymilk, peanuts) are not specifically mentioned as a food group by Swank. However, the recipes in his book [48] include legumes. Peanuts are allowed on the Swank diet if they are counted towards the daily oil allowance (see Section 4.6. Fats). Legumes are excluded on the WahlsElim diet and are typically avoided on a Paleo diet [149]. Legumes are high in lectins and are also avoided on the WahlsElim diet for this reason (see Nightshades in Section 4.2. Fruits and Vegetables). 

### 4.4. Grains 

Grains, especially whole grains, are recommended as part of a healthy diet [42] and have been associated with lower risk of diabetes and heart disease [157,158]; however, this risk may be moderated by specific genes [159] suggesting that in the future we may be able to predict who would or would not benefit from a high whole grain diet in terms of reducing risk for glucose intolerance, diabetes and obesity, a common co-morbid problem with MS. Grain free Paleo diets have been associated with greater insulin sensitivity and were more satiating per Calorie than a Mediterranean diet [160]. 

Little is known about the impact of grains on MS or the role of a gluten-free diet. A 2019 systematic review concluded there is not enough evidence to determine the effect of gluten on MS [161]. The ESPEN does not currently recommend a gluten-free diet for prevention of MS [92]. Whole grains are good fiber sources which may be beneficial for MS [11]. Higher fiber diets have been associated with lower weight, shifts in the microbiome [162] and fewer inflammatory cytokines [163]. A cross-sectional study of 6989 pwMS found individuals in the top quintile of whole grain intake had lower odds of severe disability than individuals in the lowest quintile [68]. Additional research is needed.

Swank grain guidelines are consistent with the DGA [42] in the recommendation to consume whole grain sources “as much as possible” [49]. The four grain servings per day recommended on the Swank diet does not meet the recommended servings for the DGA HEP at ≥1600 kcals (6694 kJ) [138].

Grains are typically avoided on a Paleo diet [149] because they were believed to have not been consumed by our ancient ancestors [71]. Grains are excluded on the WahlsElim diet because of their lectin content and association with neurological symptoms in genetically susceptible individuals; see Nightshades in Section 4.2. Fruits and Vegetables. In addition, wheat, rye and barley contain gluten which can also be metabolized into a morphine-like compound [164], gluten exorphins, which is associated with increased gastrointestinal symptoms and influence pain perception and behavior [164]. Wheat along with milk and eggs is also a common food trigger to Eosinophilic Esophagitis [154]. The exclusion of grains on the WahlsElim diet along with the limitation or restriction of other carbohydrate-containing food groups (e.g., legumes, dairy, added sugars, processed foods) produces a diet pattern that is lower in total carbohydrates, glycemic load and glycemic index compared to the standard American diet [70,71].

### 4.5. Dairy 

Fat-free and low-fat dairy products are included in the DGA [42]. Recommended amounts are three cup equivalents per day for the HEP at 1600–3200 kcals (6694–13,389 kJ) [138]. Dairy foods are good sources of calcium, vitamin D, potassium and vitamin A; excluding these foods significantly impacts the adequacy of calcium, vitamin D, potassium and choline in the DGA diet pattern [165]. Although fat-free and low-fat dairy products are recommended, some recent studies suggest that full fat dairy may have higher bioavailability of key nutrients, anti-inflammatory properties, have neutral effect on cardiovascular risk and that fermented dairy products such as yoghurt, kefir and cheese have a neutral or positive effect on cardiovascular risk [166,167,168,169,170].

The role of dairy products in the development and treatment of MS is unclear [11] and additional research is needed. Milk but not cheese consumption was associated with MS prevalence [171]. A recent case control study of 113 pwMS and an equal number of healthy women found a reduced risk for MS with low-fat dairy consumption > five times per week [172]. Two studies with conflicting results associating dairy with disease activity, quality of life or disability were limited by the inability to determine the type of dairy consumed [11]. After assessing the limited and conflicting evidence related to dairy and MS, authors of one editorial recommended pwMS consume low fat dairy products because of the nutrient content of these foods, especially calcium, which may reduce risk for osteoporosis [173]. 

One theory for the potential negative effect of milk in pwMS is allergic response. Milk allergy is the most frequent food allergy in children [174]. Six percent of adults with seasonal allergy have IgG antibodies to milk [153]. Cow’s milk formula is associated with higher rates of type 1 diabetes and insulin autoantibodies in infants at genetic risk for developing type 1 diabetes [175]. Milk along with eggs and wheat are common food triggers to Eosinophilic Esophagitis [154]. Casein, a milk protein, can be metabolized into Beta-casomorph-7, a morphine-like substance [176,177] which is associated with increased gastrointestinal symptoms and slower cognitive processing speed [178]. Antibodies for intestinal inflammation were elevated in schizophrenic patients compared to controls and positively correlated with IgG antibodies to the milk protein casein [179]. Butyrophilin is a dairy compound structurally similar to a compound in the brain, myelin oligodendrocyte glycoprotein (MOG), which through molecular mimicry may lead to immune mediated damage to myelin and neurons thus increasing risk of MS, cerebellar ataxia and neuropsychiatric symptoms [180]. However, in a case control study of 48 pwMS and 48 controls elevated levels of cow’s milk specific IgE antibodies were not observed [181]. 

Swank recommends consumption of fat free/low fat dairy products to minimize saturated fat intake and this is consistent with the current DGA [42]. However, the two servings per day of dairy products recommended on the Swank diet is less than the three cup recommendation for the HEP [138] at ≥1600 kcals (6694 kJ).

Dairy is typically avoided on a Paleo diet [149] and is also excluded on the WahlsElim diet, although clarified butter (ghee) is permitted because the milk solids have been removed. Dairy is avoided on the WahlsElim diet because of its potential for producing negative symptoms described above. 

### 4.6. Fats

The DGA recommend use of oils that contain a greater proportion of monounsaturated and polyunsaturated fats than saturated fat and in amounts within an individual’s energy needs [42]. Oils are good sources of essential fatty acids, vitamin E and energy for weight maintenance [42]. Acceptable Macronutrient Distribution Range (AMDR) for total fat is 20–35% of energy [182]. The DGA recommend <10% energy from saturated fat to reduce risk of cardiovascular disease; saturated and *trans-*fatty acids have been associated with higher rates of cardiovascular disease, insulin resistance and obesity [183] and should be avoided. 

Various studies investigating the effect of fat on MS have been summarized [11,141,184,185]. Based on all currently available evidence, the ESPEN recommends consuming foods lower in saturated and higher in polyunsaturated fat for prevention of MS but they do not recommend omega-3 supplements to prevent MS [92]. The ESPEN does not recommend omega-3 supplements to reduce MS relapses but suggests supplemental omega-6 fatty acids may potentially be beneficial [92]. Additional research is needed.

Saturated fats are inflammatory and may lead to gut dysbiosis [186]. Saturated fats also increase translocation of endotoxin into blood stream of mice and humans but the addition of fiber reduces this effect [163] and the addition of fish oil protects against the endotoxemia [187]. Endotoxins increase activation of the innate immune system and increased inflammatory cytokines, which in theory may have an effect on MS related symptoms. Olive oil is beneficial for neuroprotection [188]. Olive oil has 36 different phenols with health benefits. Hydroxytyrosol is the predominate phenol and has been the most studied. Hydroxytyrosol intake is associated with favorable changes in antioxidant enzyme activity and lower levels of nuclear factor *kappa*-light-chain-enhancer of activated *B* cells (NF-kappa B) activation [189,190]. However, an Australian case-control study did not find any associations between total, saturated, monounsaturated, polyunsaturated, omega-6 or plant sources of omega-3 fat and risk of first demyelinating event; total omega-3 and omega-3 fat from fish were associated with reduced risk of first event, however, fat from dietary supplements were not included in the analysis [141]. 

#### 4.6.1. Total Fat

Total fat amounts on the Swank diet are reduced [23,191] because of the restriction of saturated fat and recommendations for oil intake between 20 and 50 g per day; Swank estimated his patients consumed 25–29% of energy from total fat [23]. Swank oil recommendations are similar to DGA HEP oil amounts for 1600–3200 kcal (6694–13,389 kJ) diets [138]. In contrast, fat Calories on the WahlsElim diet are increased and could potentially be higher than the AMDR. The WahlsElim diet advises individuals to increase intake of healthy fats (i.e., non-*trans*-fat) to provide adequate energy intake. The higher fat level is required to offset the moderate protein (meat and fish) intake and reduction in total carbohydrate intake from the elimination of grains and other carbohydrate sources like grains, dairy and added sugars. 

#### 4.6.2. Saturated and Trans Fatty Acids

The main goal of the Swank diet is to limit saturated fat intake to ≤15 g per day based on his epidemiological evidence that fat from meat and dairy was associated with MS incidence. Swank estimated his patients consumed 15–17% of energy from ‘fats’ (i.e., saturated fat) [23]. This saturated fat estimate is higher than current recommendations; however, fat intake calculated with modern nutrient databases and dietary collection methods might yield different estimates. In contrast, Dr. Wahls does not restrict saturated fat intake unless it is clinically indicated. The WahlsElim diet recommends saturated animal fats (ghee, lard or duck fat) or coconut oil for cooking (frying) rather than unsaturated fats which may be damaged when heated. The WahlsElim diet includes fiber-rich fruits and vegetables and supplemental cod liver oil to help mitigate the potential negative effects of higher saturated fat intake described above. Both WahlsElim and Swank diets recommend avoiding *trans-*fats and foods containing them because of the negative health associations. 

#### 4.6.3. Monounsaturated and Polyunsaturated Fatty Acids

The Swank diet encourages mono and polyunsaturated fat intake by the use of 20 to 50 g oil per day depending on energy needs. Unsaturated fats from high fat foods such as fatty fish, nuts, avocado and olives as well as oils and salad dressings count towards the daily allotment. Use of sunflower, olive, safflower, sesame, canola, cottonseed, linseed, soybean, peanut and flaxseed oils are recommended on this diet [49]. 

The WahlsElim diet encourages use of avocado, olive, sesame and sunflower oils which are sources of monounsaturated fatty acids. Flax, walnut and hemp oils are also encouraged as a source of alpha-linolenic acid, an essential fatty acid, but amounts are limited to two tablespoons per day to maintain balance in omega 6:3 ratio. Higher alpha-linolenic acid intake was associated with reduced risk of MS in the Nurses’ Health Study II [192]. Individuals are encouraged to use all these oils, especially olive oil, cold to reduce the damage to the polyphenols caused by heating [193,194]. Heating olive oil to greater than 180 degrees Fahrenheit (82 degrees Celsius) is associated with 60% reduction of hydroxytyrosol content [193,194]. Use of oils that are cold pressed and do not require solvent extraction such as olive oil, flax oil, hemp oil and walnut oil are also preferred for the WahlsElim diet.

Individuals consuming the WahlsElim diet are encouraged to reduce intake of vegetable oils and consume grass fed meats and wild caught fish according to their financial means which will shift the omega 6:omega 3 fatty acid ratio towards the more favorable ratio of 4:1 [195,196]. Canola, corn and soybean oil are sources of omega-6 fatty acids and are to be avoided to maintain omega 6:3 ratio near 4:1 [195,196]. Canola oil is processed using solvent for extraction of the oil and should also be avoided to decrease exposure to solvents [197]. The omega 6:3 fatty acid ratio modulates neuronal membrane fluidity, neurotransmitter manufacturing and central nervous system inflammation. 

### 4.7. Sweeteners

Added sweeteners increase dietary energy, carbohydrate and glycemic index and are low in micronutrients. Added sugar intake has been associated with unhealthy blood lipid levels [198,199]. The DGA recommend limiting added sugars to <10% of energy [42] to avoid excess energy intake while meeting other food group and nutrient recommendations. 

Swank recommended limiting high sugar foods (e.g., sugar, jelly, honey, maple syrup) because they may increase nervousness ([48] p. 115). He did not prescribe a maximum amount but indicated it should be “minimal and for taste only.”

Sweeteners are either avoided or limited on the WahlsElim diet. Energy providing sweeteners are limited to reduce the diet’s carbohydrate load and glycemic index. Artificial sweeteners are avoided because they have been shown to disrupt the microbiome, decrease satiety and are associated with weight gain [200].

### 4.8. Salt and Sodium

The DGA recommend limiting sodium (Na) intake to <2300 mg per day because of the association between increased intake and higher blood pressure [42]. However, restricting salt intake for the purpose of improving MS disease course is controversial [201,202,203] and more research is needed [204]. Na intake, such as from salt, increases TH17 activity in animal models [205,206,207] but data in humans are not as clear; Na intake in humans is not predictive of MS disease activity [208]. Mechanisms of what appears to block the deleterious effect of Na on MS disease activity include potassium supplementation and higher dietary intakes from increased quantities of F/V and less processed food [209,210,211]. Compounds that block inappropriate TH17 activity include curcumin [212,213,214,215,216], vitamin D [217] and sulforaphane (cabbage family vegetables) [218]. 

Swank did not specifically recommend the use or restriction of salt or prescribe a Na intake in his book or the Swank MS Foundation website, however, cola soft drinks were restricted to 16 ounces (491 g)/day because of the Na content ([48] p. 116). He provided recipes for herb mixtures that could be used as salt substitutes ([48] p. 137) but also included salt in his recipes and high Na condiments such as soy sauce, suggesting Swank did not specifically restrict salt. However, the Na content of the diet may be lower than those of typical Americans due to the limited intake of commercial foods (see Section 4.11. Processed Foods) which are significant contributors to Na intake [42] and the elimination of salty meats such as bacon, ham, lunch meat, hot dogs and sausage due to their high fat content.

Salt is also not restricted on the WahlsElim diet. However, Paleo diets are typically lower in Na [70] because they exclude commercially processed foods that are high in Na and avoid adding salt when preparing or eating food [71,149]. In addition, Paleo diets tend to have a lower sodium:potassium ratio compared to typical American diets due to the lower Na level and the higher potassium intake from increased quantities of F/V [71]. Individuals following the WahlsElim diet who are using table salt would be advised to purchase iodized table salt or iodized sea salt to minimize risk for iodine deficiency; see Section 4.12.1. Seaweed and Algae.

### 4.9. Alcohol

The DGA maximum alcohol intake for adults who drink is one drink per day for women and two drinks per day for men [42]. Alcohol is a known neurotoxin and is associated with activation of the microglia [219] but there are no specific alcohol recommendations for pwMS. A study of 923 pwMS found moderate alcohol intake (>3 glasses red wine or >4 drinks per week) was associated with greater brain lesion volume on MRI but also with reduced disability and disease severity score [220]. Another study found regular consumption of alcohol associated with reduced disability progression in individuals with relapsing-remitting MS but not progressive MS [221]. Alcohol consumption may be contraindicated with some medications taken by pwMS such as those for sedation, pain, muscle spasms or mental health issues; pwMS should follow their doctor and pharmacist’s advice regarding alcohol use with medications. 

Swank believed his patients could tolerate half as much alcohol as they could prior to the disease ([48] p. 28) and recommended a maximum of one alcoholic beverage per day [49]. He hypothesized the reduced tolerance could be due to increased gastrointestinal motility secondary to the low fat diet which resulted in faster alcohol absorption or because of a more porous blood-brain barrier which resulted in more alcohol reaching the brain ([48] p. 28). The WahlsElim diet alcohol limits are consistent with the DGA [42] except that alcohol made from gluten-containing products is avoided to reduce gluten exposure.

### 4.10. Caffeine

According to the DGA, healthy adults can safely consume a moderate caffeine intake of three to five cups (720–1200 mL) coffee (400 mg caffeine) per day [42]. This recommendation was based on data showing coffee intake was not associated with chronic diseases [42]. Consuming three to four cups of coffee per day has been associated with decreased CVD risk [222]. Moderate caffeine and coffee consumption also does not appear to increase risk of developing MS [223] and may be associated lower risk of developing MS [224]. In another study, caffeine intake was associated with lower disability and fatigue [225]. Coffee may exert its protective effects in neurodegenerative diseases, including MS-related neurodegeneration, by blockade of adenosine receptors [226]. There are no specific caffeine intake guidelines for pwMS.

Swank restricted caffeine intake (coffee, tea, cola soft drinks) to three cups per day because he observed that it contributed to anxiety and nervousness in some of his patients which could then exacerbate other MS-related symptoms and have a negative impact on personal relationships ([48] p. 29). Swank’s caffeine restriction is consistent with DGA recommendations for healthy adults [42]. The WahlsElim diet places no restriction on caffeine intake unless the patient has difficulty with sleep, in which case patients are advised to limit the number of cups and the timing of caffeinated beverages. 

### 4.11. Processed Foods

Processed foods are sources of beneficial nutrients as well as nutrients to be limited such as saturated fat, sodium, added sugar and energy [227].

Processed foods that are high in saturated fat are avoided on the Swank diets to keep saturated fat intake ≤ 15 g per day. The WahlsElim diet also eliminates processed foods as a strategy to achieve a low added sugar and *trans*-fat intake and because these foods may contain prohibited ingredients such as grains and dairy. 

### 4.12. Other Components

#### 4.12.1. Seaweed and Algae

Dietary seaweed and algae have been recognized as being rich sources of carotenoids with significant potent antioxidant activity and potential for reducing excess and inappropriate inflammation [228,229]; see Section 4.2. Fruits and Vegetables. Seaweed is also a source of trace minerals including iodine and calcium and dietary fiber [230,231]. 

Swank did not recommend or prohibit seaweed or algae intake. These foods are low in saturated fat and would not be contraindicated on the diet. 

The WahlsElim diet encourages seaweed consumption because of the mineral composition which will help reduce the risk of deficiency. A Paleo diet has been shown to increase risk of iodine deficiency because of salt and dairy restrictions [232]; therefore, individuals on the WahlsElim diet would be advised to consume seaweed and/or purchase iodized sea salt or iodized table salt [233] to increase iodine intake. When we have insufficient nutrient mineral intake our cells may substitute other related non-nutrient minerals in their place. This would increase the risk of increased absorption of halogens [234,235] and toxic minerals [114]. Some algae may be beneficial in treating lead and cadmium toxicity [236]. The cell walls may chelate heavy metals that are excreted in the bile [114]. According to the National Multiple Sclerosis Society, heavy metals do not cause MS [237], however, a case report documented improvement in MS symptoms following chelation therapy [238]. Mercury exposure is associated with increased risk of autoimmune dysfunction [239], oxidative stress and neurodegeneration [240]. The WahlsElim diet encourages a mixture of algae and seaweeds for diversity. Chlorella is the preferred form of algae due to detection of cyanotoxin contamination of some commercially available spirulina based products [241]. Avoid wild type blue green algae to decrease the risk of contamination from wild algae associated with toxic algae blooms [242]. 

#### 4.12.2. Nutritional Yeast

Nutritional yeast is an excellent source of B vitamins. One tablespoon provides [243] >300% Daily Value (DV) vitamins B1 and B2, >200% DV vitamin B6, >100% DV vitamin B3, 73% DV vitamin B12 and 33% DV folate. B vitamins are key nutrients for mitochondria and ATP energy production and improved neural repair [244]. 

Nutritional yeast is low in saturated fat and would be permissible on the Swank diet. Swank did not recommend regularly consuming a specific amount of nutritional yeast but he did suggest a glass of skim milk with one tablespoon (9 g) nutritional yeast as an afternoon snack option ([48] p. 123). 

Regular consumption of nutritional yeast is encouraged on the WahlsElim diet to boost the B vitamin content of the diet. Nutritional yeast also offers individuals a foodstuff with a cheese like flavor for those who miss cheese in their diet. 

#### 4.12.3. Fermented Food

Fermented foods include tea (kombucha), vegetables (sauerkraut, olives, pickles, kimchi and other fermented vegetables), dairy (yogurt, kefir, raw cheese), soy (natto, miso, tempeh), wine and beer, meat (sausage) and bread [245]. Consumption of various fermented foods have been associated with positive effects related to type 2 diabetes, cardiovascular disease, obesity and other conditions and can increase the number of potentially beneficial microbes in the diet [245]. Evidence grows that the gut microbiome of pwMS differs from healthy individuals [246,247,248,249]. Diet changes in a mouse model of MS altered gut microbes and impacted disease severity [250]. 

The Swank diet does not mention fermented foods, however, the recipes in his book include fermented food in the form of nonfat yogurt [48]. Fermented foods would be permitted on the Swank diet if they are low in saturated fat and if the caffeine-containing kombucha is counted towards the three cups of caffeine allowed per day. Regular consumption of fermented foods that contain live lactobacillus organisms [251] and are made from allowed foods [252] is encouraged on the WahlsElim diet for their potential favorable impact on the gut microbiome. 

### 4.13. Dietary Supplements

Swank and WahlsElim diets recommend different dietary supplements (Table 1). The randomized, controlled trial [45,46] comparing the two diets prescribes the WahlsElim supplements to both Swank and WahlsElim diet groups to eliminate differences in nutrient intake due to supplements.

#### 4.13.1. Cod Liver Oil

Cod liver oil is a source of pre-formed vitamin A, vitamin D, EPA and DHA all of which are anti-inflammatory [186]. Vitamin A is needed for activation of the anti-inflammatory properties of vitamin D [186] and induction of regulatory T cells [253] which may make it superior to a fish oil supplement. More recent research has demonstrated higher omega 3 fatty acid intake is associated with lower risk of developing MS [192] and the first demyelinating event [141]. However, with the limited evidence, ESPEN clinical guidelines do not currently recommend supplementation with omega-3 fatty acids to prevent MS or reduce MS relapse [92].

Swank recommended five grams cod liver oil beginning with his paper in 1953 [20]. He did not provide the rationale for the recommendation in this paper; however, his book and website state it provides unsaturated fatty acids and energy, reduces colds and flu and provides vitamins A and D [48,49]. Swank’s 50 year report recommended vitamin A and D capsules to prevent deficiency [26]. Since cod liver oil is also a source of vitamin A and D he may have prescribed cod liver oil to prevent deficiencies in these nutrients. The WahlsElim diet also recommends five grams cod liver oil daily to provide EPA, DHA and vitamins A and D. If patients regularly consume organ meat, the cod liver oil could be replaced with fish oil. Cod liver oil should be refrigerated; if refrigeration is not available, capsules can be taken.

#### 4.13.2. Multivitamin/Mineral

In his first report, Swank recommended a multivitamin supplement that provided vitamins A, D, C and B complex [20]. His 50 year report recommended a multivitamin to prevent deficiencies [26]. Today, a multivitamin/mineral supplement is recommended with a limit of one capsule per day to avoid excessive vitamin A and D intake given that cod liver oil is also a source of these fat soluble vitamins [48,49]. The WahlsElim diet also recommends a multivitamin/mineral supplement but prescribes one without iron, even for women of childbearing years, because the WahlsElim diet is unlikely to be deficient in iron and iron excess is associated higher risk of neurodegeneration [254]. 

#### 4.13.3. Vitamins C and E

Swank recommends 1000 mg vitamin C and 400 IU vitamin E in addition to the amounts found in the multivitamin/mineral supplement [49] because of the antioxidant capabilities of these two nutrients which prevents oxidation of unsaturated fats [48]. His book initially recommended 25 to 50 IU vitamin E when consuming cod liver oil and high intakes of fish and polyunsaturated fat, an amount found in many therapeutic multivitamin supplements ([48] pp. 105, 106, 125). The WahlsElim diet does not recommend additional vitamin C and assumes the F/V intake and multivitamin/mineral will provide a sufficient amount of this nutrient. The WahlsElim diet also does not prescribe additional vitamin E beyond what is found in the multivitamin/mineral supplement. 

#### 4.13.4. Methylfolate and Methylcobalamin

The WahlsElim diet recommends 1000 mcg of methylfolate and 1000 mcg of methylcobalamin. Elevated homocysteine levels are a reflection of folate and cobalamin status and are associated with increased risk of neurodegeneration [255]. Methylated forms of folate and cobalamin are used to improve methylation and decrease the risk of hyperhomocysteinemia. Although there is a 1000 mcg upper limit (UL) for synthetic folic acid to prevent neuropathy in individuals deficient in cobalamin, there is no UL for folate naturally occurring in food [256]. Patients are encouraged to work with their medical team to have their homocysteine level closely monitored and methylfolate and methylcobalamin doses adjusted to achieve a homocysteine level between 4 and 7.5 mmol/L [85]. Swank does not recommend supplemental folate or vitamin B12 beyond what is in the multivitamin/mineral preparation which typically provides amounts that meet the Recommended Dietary Allowances.

#### 4.13.5. Vitamin D3

Vitamin D is synthesized in humans by ultraviolet light activation of cholesterol molecules in dermis layer of the skin. Because of societal changes in work and recreation, many persons have decreased exposure to sunlight which may contribute to lower vitamin D blood levels. MS patients with vitamin D levels <40 ng/mL have a higher risk of relapse and enhancing lesions on MRI [257]. However, the ESPEN did not believe there was enough evidence to recommend vitamin D treatment for the purpose of reducing relapses; they do recommend having sufficient vitamin D levels for the prevention of MS [92].

Swank did not recommend a vitamin D supplement beyond what is found in the cod liver oil and multivitamin. The WahlsElim diet, however, recommends 5000 IU (125 mcg) vitamin D3 as an initial dose with close monitoring of vitamin levels by the personal physician to achieve a level of 40–80 ng/mL based on studies correlating lowest disease activity with blood levels of >40 ng/mL [257]. Although the initial dose exceeds the 100 mcg (4000 IU) Vitamin D UL set by the Institute of Medicine [258], the amount is lower than the 250 mcg (10,000 IU) dose recommended to correct low blood levels [259]. 

## 5. Comparison of Swank and WahlsElim Diets

The Swank and WahlsElim diets were developed to treat MS based on research current at the time, however, their eating plans differ in grain, dairy, fat/saturated fat intake and quantity of F/V. Swank’s key diet strategy was reducing fat and especially saturated fat consumption but his diet is also largely consistent with the 2015–2020 DGA HEP for healthy eating except for a lack of specificity for intake of vegetable subgroups (e.g., leafy, red/orange), and slightly lower quantity of vegetables, grains and dairy. The WahlsElim diet, on the other hand, which generally follows Paleo diet principles, excludes whole food groups (grains and dairy) and allows unlimited saturated fat intake which is not consistent with the DGA. Other key differences are the large quantity (nine+ cups) of F/V the WahlsElim diet recommends which is more than the amount included in the DGA HEP and the inclusion of special dietary components (e.g., seaweed, nutritional yeast, fermented food) which provide substances Dr. Wahls believes are important for neuronal health.

Interestingly, both Swank and Wahls believe their diets are consistent with ‘healthier,’ whole food diets eaten in earlier times. According to Swank, our ancestors ate less fat than today due to its unavailability and high cost and did not consume processed ‘junk’ food because it did not exist ([48] p. 4). Swank attributed his patients’ good outcomes to the reduced fat intake but some believed it was due to the overall healthy eating style ([48] p. X). For Dr. Wahls, returning to the nutrient-dense diet of our earliest ancestors [69,70,71] was a key factor driving her diet design because adequate intake of many nutrients have been associated with brain health [14,15]. 

In addition to the similar perceived healthfulness of both diets, Swank and Wahls also found that individuals with less severe disease who adopted their diet earlier in their disease course and who adhered more closely to the diet had better outcomes. A recent international study of 2138 pwMS found increased odds of fatigue with a poor diet [260]; intake of fish ≥ three times a week, use of flaxseed oil (omega-3 fatty acid), moderate alcohol consumption, vitamin D supplements and exercise were associated with reduced odds for fatigue [260]. These dietary findings are consistent with both Swank and Wahls™/WahlsElim diets which recommend fish, cod liver oil (vitamin D and omega-3 source) and moderate alcohol. A 2018 survey of 6989 pwMS found reduced disability associated with better diet quality defined as increased intake of F/V and whole grains and reduced consumption of sugars and red and processed meat; an overall healthy lifestyle which included a healthy diet was also associated with less severe fatigue [68]. Individuals following the Wahls™ diet now or in the past had greater disability as measured by the Patient-Determined Disease Steps questionnaire than individuals who were not following the Wahls™ diet. However, a greater proportion of individuals with progressive MS chose to follow the Wahls™ diet than other diets; the authors speculated that these individuals had more significant disability and chose to follow this diet in an attempt to treat their more severe symptoms [68]. Additionally, the study found no association between disability and individuals who followed the Swank diet. Individuals who had higher intake of whole grains and dairy had less disability; this would seem to favor the Swank diet which includes these food groups over the Wahls™ diet which excludes them. A 2018 systematic review concluded that additional research is needed to determine the effect of diet on MS-related fatigue [261]. 

Swank and Wahls also found that individuals following their diets tended to lose weight. A 2015 study of pwMS found increased odds of fatigue with obesity [260]. Additionally, following a weight loss diet has been associated with lower disability [68]. 

Although Swank and Wahls consider their diets to be ‘healthy,’ the nutritional adequacy of their eating plans has not been rigorously evaluated. Diets that exclude entire food groups such as WahlsElim potentially increase the probability of nutritional insufficiency but Dr. Wahls and other Paleo diet researchers believe the Paleo and WahlsElim eating patterns are nutrient dense even though they do not comply with the healthy eating recommendations promoted by the DGA. A nutritional analysis of the Swank diet and WahlsElim diet would determine if there are gaps in specific nutrients which should be addressed. Both Swank and Wahls published books for the lay public that include menus and recipes which can be analyzed for nutritional adequacy. This analysis has been completed and is being submitted for publication.

Free living individuals may select different recipes and meal plans than listed in the books so it will be useful to also conduct an analysis of the diet consumed by pwMS to understand the diet quality of individuals who follow the Swank and Wahls™/WahlsElim diet plans. One limited report of single 24 h dietary recall data from two individuals following each diet found nutrient deficiencies in both diets [67]. Participants in The Dietary Approaches to Treating Multiple Sclerosis Related Fatigue Study (NCT02914964) [45] will have the quality of their baseline usual diet and randomized study diet assessed using the Harvard Food Frequency Questionnaire and three day weighed food records [46]. Study completion is anticipated in 2019 with analysis of the data to be completed in 2020. 

## 6. Conclusions

Environmental factors including diet quality may impact the development and severity of MS symptoms. The Swank and Wahls™ diets have case series and pilot data showing favorable impact on MS symptoms. However, the studies did not include a control group. Larger, better quality, randomized controlled trials are needed to investigate the impact of these diets on MS-related fatigue and QOL. A dietary intervention study comparing the efficacy of the Swank and WahlsElim diet for reducing MS-related fatigue is underway and will include an analysis of diet quality. Further research on the impact of diet quality on MS symptoms is needed, including intervention studies and longitudinal observational studies that include clinical outcomes and nutrition data. As insights into the role of specific nutrients and the role microbial metabolites in neurodegenerative and neuroinflammatory disease expands, new theoretic dietary recommendations for pwMS will likely be developed and evaluated. 

## Figures and Tables

**Table 1:** Low saturated fat Swank diet [48,49] and modified Paleolithic Wahls Elimination [46] diet guidelines.

Diet Component	Swank Diet	Wahls Elimination Diet
Calories (kilojoules)	Adjust to meet energy needs	Adjust to achieve and maintain a healthy body mass index
Fruits and Vegetables	2+ cup-eq ^1^ (~45–250 g) fruit per day, fresh preferred ^2^	2–3+ cup-eq (~60–420+ g) ^3^ dark green leafy vegetables per day
	2+ cup-eq (~20–250 g) vegetables ^2,4^ per day	2–3+ cup-eq (~40–765+ g) ^3^ sulfur-rich vegetables ^5^ per day
		2–3+ cup-eq (~40–765+ g) ^3^ deeply colored fruits and vegetables ^6^ per day
		White fruits and vegetables limited ^7^
		Nightshade vegetables/spices avoided ^8^
Protein Foods	Adequate in quantity and quality	6–12+ ounces (170–340+ g) meat/fish per day
- Beef, Pork, Poultry, Game, Fish	Beef, pork and dark meat poultry not allowed the first year ^9^; white meat poultry without skin allowed as desired; ≤ 1.75 ounces (50 g) fatty fish/day ^10^; white fish and shellfish as desired	Beef, pork, poultry and game allowed as desired; 16 ounces (454 g) omega-3 rich fish/week encouraged; other fish and shellfish as desired
- Organ Meat	Not allowed during first year ^9^	12 ounces (340 g)/week encouraged
- Eggs	Whole eggs ≤1/day, ≤3/week; egg whites as desired	Not allowed
- Nuts	Allowed in limited amounts ^10^	Maximum 4 ounces (113 g)/day, soaked and rinsed
- Legumes ^11^	Allowed if low in saturated fat ^10^	Not allowed
Grains	4 servings ^12^ grains/day, whole preferred ^2^	Not allowed
Dairy	2 cups (490 g ^13^) dairy products with <1% fat per day	Cow, goat, mare and soy products not allowed
Fats		As desired for satiety and weight maintenance
- Saturated	≤15 g saturated fat/day	Clarified butter ^14^, animal fats ^14^, coconut oil^14^, coconut milk as desired; butter and *trans* fats not allowed
- Unsaturated	4 to 10 teaspoons (20–50 g) oil ^10^/day	Avocado oil, extra virgin olive oil, sesame oil, sunflower seed oil allowed as desired; flax, hemp and walnut oil, maximum 2 tablespoons (30 g)/day; all other fats and oils not allowed
Sweeteners	Minimal use for taste	Allowed sweeteners ^15^ ≤ 1 teaspoon (4–7 g)/day
Salt	As desired ^16^	As desired
Alcohol	1 drink/day (wine, mixed drink in evening)	≤1 drink/day women; ≤2 drinks/day men
Caffeine	Caffeinated beverages ≤ 3 cups (237–246 g)/day	No restriction
Processed Foods	Products containing saturated fat or hydrogenated oils not allowed	Products containing *trans*-fat and other non-approved ingredients not allowed
Other		
- Seaweed/Algae	Neither prohibited or encouraged	1 serving ^17^ seaweed and 1 serving ^18^ algae encouraged daily
- Nutritional Yeast	Neither prohibited or encouraged	1 serving ^19^ encouraged daily
- Fermented Food ^20^	Neither prohibited or encouraged; must be low in saturated fat	1 serving ^21^ encouraged daily
Supplements ^22^	1 teaspoon (5 g) cod liver oil	1 teaspoon (5 g) cod liver oil
	1 multivitamin/mineral	1 multivitamin/mineral for men 50+ years
	1000 mg vitamin C^2^	1000 mcg methylfolate
	400 IU vitamin E^2^	1000 mcg methyl B12
		5000 IU (125 mcg) vitamin D3 ^23^

^1^ cup equivalents; 2 cups raw leafy (~30–140 g), 1 cup raw or cooked (~35–250 g), 1 cup juice (~245–250 g), ½ cup dried (~20–90 g); ^2^ Swank MS Foundation [49] updated these guidelines after *The Multiple Sclerosis Diet Book* [48] was published; ^3^ quantity adjusted depending on appetite and energy needs; ^4^ Avocado and olives count towards daily oil allotment; ^5^ Includes cruciferous (e.g., broccoli, cauliflower) and allium vegetables (e.g., garlic and onions) as well as mushrooms; ^6^ Includes carrots, beets, sweet potatoes, cherries, berries and similar fruits/vegetables with color throughout; a variety of colors including red, blue/black/purple, green and yellow/orange are encouraged; ^7^ Includes apples, pears, bananas; allowed after recommended leafy, sulfur and color requirements are met; ^8^ tomato, white potato, eggplant, peppers; may be re-introduced to the diet after three months if well tolerated; ^9^ May be reintroduced after one year and limited to 3 ounces (85 g) per week; ^10^ Unsaturated fat in fatty fish and nuts (including the legume peanuts) are counted as part of the oil allowance; ^11^ Includes dried beans and peas, green beans, soy products, peanuts; ^12^ Serving = one ounce (28 g) equivalent such as 1 slice (28g bread), ½ cup cooked rice (79 g); ^13^ Weight for 2 cups fluid milk or yogurt; ^14^ Saturated fats are for high heat cooking; ^15^ Honey, maple syrup, molasses, sugar allowed; artificial sweeteners, sugar alcohols, high fructose sweeteners not allowed; ^16^ Swank did not prohibit or encourage salt intake, however, cola soft drinks were restricted to 16 ounces (491 g)/day because of the sodium content ([48] page 116); ^17^ Serving = ¼ tsp dried powder (0.5 g); ^18^ Serving = 1/2 tsp dried powder (1 g); ^19^ Serving = 1 tablespoon (9 g); ^20^ Includes yogurt, kefir, kombucha, sauerkraut, pickles, kimchi, natto, miso and tempeh; ^21^ Serving = ¼ cup fermented vegetable (~80–210 g), ½ cup kombucha tea (122 g); ^22^ In the clinical study [46], individuals randomized to the Swank diet receive the same supplements as Wahls Elimination diet to eliminate supplement differences between diets; ^23^ dose adjusted based on blood level.
